# Dialysis Disequilibrium Syndrome: Rare Serious Complication of Hemodialysis and Effective Management

**DOI:** 10.7759/cureus.5000

**Published:** 2019-06-25

**Authors:** Sreedhar Adapa, Venu Madhav Konala, Narothama Reddy Aeddula, Vijay Gayam, Srikanth Naramala

**Affiliations:** 1 Nephrology, The Nephrology Group, Visalia, USA; 2 Internal Medicine/ Hematology and Oncology, Ashland Bellefonte Cancer Center, Ashland, USA; 3 Medicine & Nephrology, Deaconess Health System/ Indiana University School of Medicine, Evansville, USA; 4 Internal Medicine, Interfaith Medical Center, New York, USA; 5 Rheumatology, Adventist Medical Center, Hanford, USA

**Keywords:** dialysis, seizures, dialysis disequilibrium syndrome, cerebral edema

## Abstract

Dialysis disequilibrium syndrome (DDS) is an extremely rare central nervous system complication that occurs in patients receiving hemodialysis and can be potentially fatal. The prognosis is poor when this manifests with serious neurological manifestations like seizures and obtundation. Evidence in effective management is sparse. Herein, we present the case of a patient who developed seizures and altered mental status during hemodialysis. The patient successfully recovered with the administration of mannitol and 3% hypertonic saline without any long-term neurologic sequelae.

## Introduction

Dialysis disequilibrium syndrome (DDS) is the clinical condition of acute neurologic deterioration attributed to dialysis treatment. DDS is a serious complication that occurs during or immediately after aggressive initial hemodialysis but can also develop in patients undergoing chronic dialysis. This phenomenon was first reported in 1962, but DDS with severe neurological symptoms such as mental status changes, seizures, and coma are rarely encountered in current clinic practice [1]. DDS with milder symptoms including nausea, vomiting, headaches, fatigue, and restlessness can still be evident.

## Case presentation

**Figure 1:**
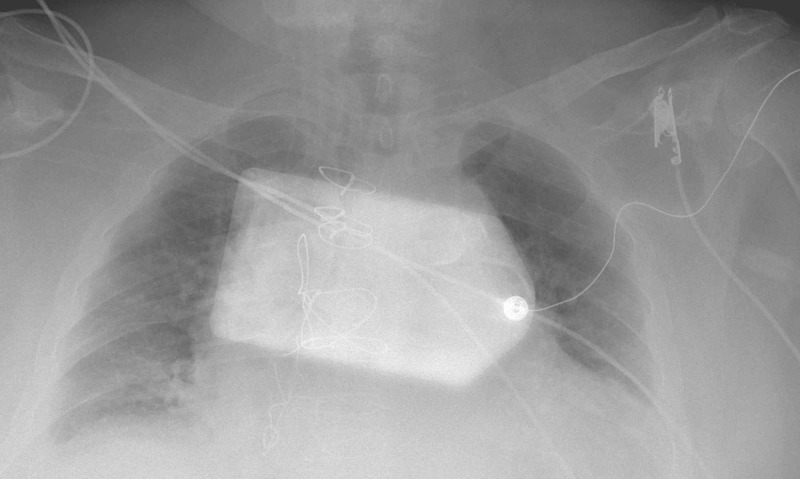
Chest X-ray single frontal view showing cardiomegaly with pulmonary venous congestion

A 72-year-old Hispanic male who missed dialysis for two weeks because of peritoneal dialysis catheter malfunction was admitted with shortness of breath secondary to fluid overload. Vitals at presentation were temperature 96 °F, heart rate 53 beats per min, respiratory rate 24 per min, blood pressure 88/60 mm of Hg, and pulse oximetry 93% on 6L nasal canula. He was hyperkalemic and bradycardic, and emergent hemodialysis was arranged with the central venous catheter (CVC) after medical management. Labs revealed severe azotemia with blood urea nitrogen (BUN) 141 mg/dl, creatinine 9.8 mg/dl, potassium 8.3 mmol/L, sodium 135 mmol/L, bicarbonate level of 11 mmol/L, and blood glucose 108 mg/dl. Chest X-ray showed pulmonary venous congestion, as shown in Figure 1. The patient was initiated on hemodialysis with low blood flow rate (BFR) 200 ml/min, and low dialysate flow rate (DFR) of 400 ml/min using a small dialyzer. The patient developed twitching all over the body and became confused about 90 minutes into hemodialysis. On neurological examination, the patient was lethargic, unable to follow commands, and randomly moving all extremities; pupils were equal reacting to light bilaterally. He was protecting his airway and did not require intubation. Hemodialysis was immediately terminated, and the patient was given mannitol 125 mg and 3% hypertonic saline was started at 30 cc/hr intravenously. Postdialysis labs revealed BUN 64 mg/dl (urea reduction ratio [URR] 54.6%), creatinine 5.2 mg/dl, and potassium 3.2 mmol/L. He had to be dialyzed again the following day for hyperkalemia (5.7 mmol/L) and fluid overload state. The patient was dialyzed with low blood and dialysis flow rates. Mannitol and 3% hypertonic saline were administered during dialysis. Computed tomography of the head did not show any acute intracranial process. Electroencephalogram revealed no epileptiform abnormality and diffuse background slowing. The patient’s neurological status gradually improved with full recovery in four days.

## Discussion

DDS usually occurs during or immediately after patients receive their first hemodialysis treatment but can rarely happen in patients on chronic dialysis [2-3]. DDS is more common in children than adults and is more frequently associated with hemodialysis than peritoneal dialysis. The risk factors for developing DDS include young age, severe uremia, marked reduction of urea on initial dialysis, dialysis with ultrafiltration, low dialysate sodium concentration, high flux and large surface area dialyzers, and preexisting neurological disorders [2-3].

Dialysis removes osmotically active molecules like urea from the blood more rapidly than they can diffuse out of the brain causes osmotic gradient leading to the flow of water into the brain, with the end result of cerebral edema. Ronco et al noticed changes in brain density on CT scan during and after hemodialysis which postulates that there might be postdialysis gain in cerebral water [4]. Although the exact pathogenesis of DDS is still debatable, multifactor pathophysiological mechanisms have been postulated like reverse urea effect, accumulation of idiogenic osmoles and paradoxical acidosis in the cerebrospinal fluid [2,5]. There might be a brief urea gradient created between plasma and cerebrospinal fluid during dialysis, which can be aggravated in situations like uremia. Animal studies have shown that there is increased expression of aquaporin channels that facilitate an increased inflow of water into the brain by osmosis and decreased expression of urea transporters, which results in a decreased outflow of urea [6]. Reverse urea effect theory has been disputed and the use of urea containing dialysate had failed to prevent cerebral edema. Rapid correction of acidosis during dialysis will cause paradoxical acidosis in CSF from the diffusion of carbon dioxide across the blood-brain barrier, which is produced from bicarbonate. Elevated arterial carbon dioxide partial pressures can alter cerebral autoregulation and increase in brain osmole quantity causing cerebral edema and increased intracranial hypertension. Intracranial accumulation of idiogenic osmoles or middle molecules contributing to DDS is questionable. Changes were observed in the cerebral concentration of choline-containing compounds, myoinositol, and water in the brain on short echo-time magnetic resonance (MR) spectroscopy during dialysis [7].

DDS is a clinical diagnosis. Other conditions which can mimic DDS, such as hypoglycemia, excessive ultrafiltration, malignant hypertension, and uremic encephalopathy needs to be ruled out. DDS is a diagnosis of exclusion. Patients usually develop symptoms of DDS during or towards the end of dialysis but can be delayed up to 24 hours. The most common manifestations are nausea, vomiting, headache, blurry vision, restlessness, fatigue, muscle twitching, tremor, and hypertension. More severe manifestations which are rare include seizures, altered mental status, coma, and death [3]. Seizures are usually transient. Death may occur from central herniation. Before attributing the symptoms to DDS, the other possible causes of altered mentation should be excluded. DDS is usually self-limiting, lasting for several hours. Full recovery may take several days. Cerebral edema is only consistent finding on brain imaging; other diagnostic studies including electroencephalography are non-specific.

The management of DDS is primarily preventative. It is important to identify the patients who are at a high risk of developing DDS and use low-efficiency dialysis with gradual urea reduction. In the above scenario, our patient likely was at risk for DDS considering severe azotemia that is a surrogate for uremic milieu. Cautious reduction in the blood urea concentration by 40% would be a reasonable approach for a first dialysis session [3]. Once DDS develops, treatment is directed to reduce intracranial pressure with mannitol and hypertonic saline [3]. Our patient did well with the administration of 3% hypertonic saline and mannitol with full neurological recovery.

## Conclusions

Prognosis of severe DDS is very poor, and hence, prevention is the key in the management of DDS. There is not much guidance in literature as far as management of DDS with severe neurologic manifestations is concerned. The management of this condition in the contemporary era is extremely challenging considering the rarity of the phenomenon. These agents may have a role in the management of patients who develop DDS despite the use of preventative measures such as low-efficiency short hemodialysis sessions.
